# Comparative clinical outcomes of polymyxin-based versus non-polymyxin regimens as definitive therapy in Carbapenem-resistant *Klebsiella pneumoniae* bacteraemia

**DOI:** 10.1371/journal.pone.0353799

**Published:** 2026-07-15

**Authors:** Divya Bhat, Asha K. Rajan, Vandana Kalwaje Eshwara, Muralidhar Varma, Shashikiran Umakanth, Girish Thunga, Vishal Shanbhag

**Affiliations:** 1 Department of Critical Care Medicine, Kasturba Medical College, Manipal Academy of Higher Education, Manipal, Karnataka, India; 2 Department of Pharmacy Practice Manipal College of Pharmaceutical Sciences, Manipal Academy of Higher Education, Manipal, Karnataka, India; 3 Department of Microbiology, Kasturba Medical College, Manipal Academy of Higher Education, Manipal, Karnataka, India; 4 Department of Infectious Diseases, Kasturba Medical College, Manipal Academy of Higher Education, Manipal, Karnataka, India; 5 Department of General Medicine, Dr. TMA Pai Hospital, Udupi, Karnataka, India; University of Perugia Department of Medicine: Universita degli Studi di Perugia Dipartimento di Medicina, ITALY

## Abstract

**Background:**

Carbapenem-resistant *Klebsiella pneumoniae* (CRKP) bacteraemia poses a major therapeutic challenge due to limited effective therapeutic options and high mortality. Although polymyxins remain widely used, emerging evidence suggests that non-polymyxin regimens may offer improved efficacy and safety. This study compared clinical and microbiological outcomes between polymyxin-based therapy (PBT) and non-polymyxin regimens (NPR) in patients with CRKP bloodstream infections (BSI).

**Methods:**

In this multicentric, retrospective observational study, adult patients (≥18 years) with confirmed CRKP bacteraemia admitted between January 2019 and December 2023 were included. Patients were categorised based on definitive therapy as PBT or NPR. Baseline characteristics, disease severity, and outcomes were compared using appropriate statistical tests. Predictors of in-hospital mortality were identified by multivariable Cox regression, validated by bootstrap resampling, and assessed through landmark sensitivity analysis excluding early deaths (<48 hours). Kaplan-Meier survival analyses were performed for mortality and microbiological clearance.

**Results:**

Of 1,009 patients with *K. pneumoniae* bacteraemia, 244 patients with CRKP met inclusion criteria (PBT, n = 143; NPR, n = 101). Baseline demographics were similar, but PBT recipients had higher SOFA (4[0–12] vs.3[0–12]; p < 0.001) and CCI scores (4[0–8] vs.3[0–10]; p = 0.025). Clinical cure was achieved more often with NPR (58.4%vs.15.4%; p = 0.02), while in-hospital mortality (38.6%vs.68.5%; p < 0.001) and acute kidney injury (28.7%vs.55.9%; p = 0.008) were significantly higher in the PBT group. Microbiological clearance and time to clearance were comparable. Independent predictors of mortality included treatment with polymyxins (aHR:1.595;95%CI:1.392–1.903;p = 0.015), SOFA score>6 (aHR:1.514;95%CI:1.285–1.928;p = 0.027), history of chronic liver disease (aHR:2.31;95%CI:1.884–3.642;p = 0.02), post-therapy dialysis (aHR:3.11;95%CI:1.008–4.59;p = 0.048), and requirement of ICU admission after definitive therapy (aHR:1.474;95%CI:1.252–1.891;p = 0.02). Survival analysis confirmed superior outcomes for NPR (log-rank p < 0.001).

**Conclusion:**

NPR was associated with significantly higher clinical cure, lower nephrotoxicity, and reduced mortality compared with PBT regimens. These findings suggest that non-polymyxin regimens may be associated with improved clinical outcomes and lower nephrotoxicity compared with polymyxin-based therapy in patients with CRKP bacteraemia. Prospective studies were warranted to confirm these observations.

## 1. Introduction

Bacteraemia remains a critical global health challenge, contributing to substantial morbidity, mortality and economic burden worldwide [1–3]. Progression to sepsis is a major concern, affecting more than 30 million people annually and prompting the World Health Organization (WHO) to designate sepsis as a global health priority [4–7]. In recent years, Gram-negative bacteria have emerged as a predominant pathogen in bloodstream infections, disproportionately affecting elderly and immunocompromised populations [8–10]. The situation is further aggravated by the rise of multidrug-resistant Gram-negative bacteria (MDR-GNB), which are associated with mortality rates as high as 47% to 66% [11–13]. Increasing carbapenem use, particularly across South and Southeast Asia, has contributed to the alarming emergence and spread of carbapenem-resistant Enterobacterales (CRE) [14,15],whose prevalence in bacteraemia has more than doubled over the past two decades, from 6.3% in 1997 to 15.8% in 2016 [1,9]. Recognizing this trend, the WHO has designated CRE as a “critical priority pathogen” requiring urgent global attention [16,17].

Among CRE, *Klebsiella pneumoniae* is the most frequently encountered carbapenemase-producing Enterobacterales (CPE) [12,18]. This opportunistic pathogen causes a wide spectrum of invasive infections, including liver abscesses, sepsis, pneumonia, urinary tract infections (UTIs), necrotizing fasciitis, and meningitis [11,19]. Its resistance mechanisms, spanning carbapenemase production (KPC, NDM, OXA-48, VIM, IMP), porin loss, efflux pump overexpression and plasmid-mediated co-resistance, render many β-lactam antibiotics ineffective [20,21]. The co-circulation of strains producing multiple carbapenemase and diverse sequence types further limits therapeutic options [22]. These challenges are compounded in resource-constrained settings, where limited diagnostic capacity delays pathogen identification and resistance detection, often leading to suboptimal empirical therapy [23].

Historically, polymyxins—colistin and polymyxin B—have served as last-line therapeutic agents for CRE infections, frequently combined with aminoglycosides, tigecycline or fosfomycin to enhance bacterial killing through synergistic effects [24–28]. Despite their broad activity against MDR-GNB, polymyxins are limited by suboptimal pharmacokinetics, narrow therapeutic margins, and dose-dependent nephrotoxicity and neurotoxicity [29–31]. Accordingly, the Indian Council of Medical Research (ICMR) discourages polymyxin use as first-line therapy for CRKP infections and recommends their use only when alternative options are unavailable, ideally in combination with agents demonstrating in vitro susceptibility [32]. Nonetheless, due to cost constraints and limited access to newer antimicrobials, polymyxins continue to play a central role in the management of CRKP infections in many low and middle-income countries [33].

The introduction of newer β-lactam/β-lactamase inhibitor combinations has significantly expanded the therapeutic armamentarium against CRE. The Infectious Diseases Society of America (IDSA) 2021 guidelines recommend ceftazidime-avibactam (CAZ-AVI) as the preferred first-line option for infections caused by KPC- and OXA-48 producing strains, while other alternatives—such as cefiderocol, tigecycline, eravacycline and aminoglycosides—may be considered based on local susceptibility patterns and infection site [34,35]. Tigecycline and fosfomycin retain activity against many CRKP isolates; however, the ICMR advises against tigecycline monotherapy for bacteraemia due to low serum concentrations [32,36]. Importantly, CAZ-AVI lacks activity against metallo-β-lactamase (MBL) producers such as NDM and VIM [19] but combining it with aztreonam provides effective coverage by protecting aztreonam from hydrolysis by co-produced serine β-lactamases [23,37]. This combination (CAZ-AVI plus aztreonam) is of relevance in India, where co-production of NDM and OXA-48 is frequent [38]. While meropenem/vaborbactam and imipenem/relebactam remain ineffective against MBLs [39,40], CAZ-AVI with aztreonam is currently the preferred regimen for MBL-producing CRKP infections [27,32].

Although international studies have characterized treatment outcomes for CRKP bacteraemia [14,26,37,41], data from India remain scarce and largely limited to small, single-centre studies [23,42]. Given the evolving regional resistance patterns and limited availability of newer agents, robust local evidence is crucial to inform therapeutic decision-making. The present multicentre, retrospective observational study represents the largest cohort to date comparing polymyxin-based versus non-polymyxin regimens in patients with CRKP bacteraemia. This study aims to evaluate and compare clinical outcomes—including mortality trends—across treatment groups to provide region-specific insights that may guide optimal management strategies for CRKP infections.

## 2. Methods

### 2.1 Ethical approval

This study complied with the ethical standards of the Declaration of Helsinki and received approval from the Institutional Ethics Committee (IEC approval no:5/2024). Owing to its retrospective, non-interventional design, the requirement for informed consent was waived. The medical records used in this study were accessed from 1/07/2024–30/04/2025. All data were retrieved from institutional electronic health records by trained investigators. To maintain patient confidentiality, identifiers were removed immediately upon extraction, and only anonymized data were used for analysis in accordance with institutional and national data protection standards.

### 2.2 Study design, setting and patient population

This retrospective, multicentre, observational study included adult patients (≥18 years) with microbiologically confirmed CRKP bloodstream infections admitted to participating tertiary care hospitals in India between January 2019 and December 2023. Only the first episode of CRKP bacteraemia per patient was considered to avoid duplication.

**Fig 1:**
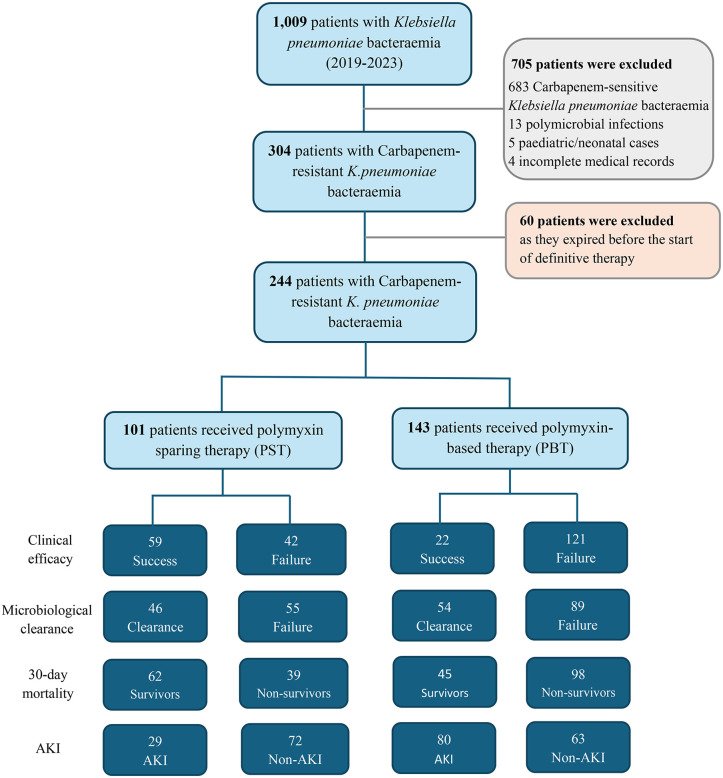
Overall methodological workflow of the study.

Patients were excluded if they i. had polymicrobial bloodstream infections, (i.e., co-isolation of CRKP with other bacterial or fungal pathogens), ii. died before receiving any definitive anti-CRKP therapy, iii. were paediatric or neonatal cases, iv. Had incomplete medical records, or v. were discharged against medical advice. Eligible records were extracted and compiled into a unified study database. Fig 1 provide a schematic overview of the methodological workflow.

### 2.3 Definitions

Carbapenem-resistant *K. pneumoniae* (CRKP) was defined as any isolate exhibiting resistance to at least one carbapenem (imipenem or meropenem) on phenotypic susceptibility testing [43]. Primary CRKP bacteraemia referred to cases where CRKP was the sole and initial bloodstream pathogen identified during hospitalization, with no preceding bacteraemia. Microbiological clearance was defined as the first negative blood culture obtained after the initial CRKP-positive culture, and time to clearance was calculated as the number of days between the initial CRKP positive culture and the subsequent first negative blood culture [44].

Treatment regimens were classified as follows, based on standard guideline suggestion [45,46]:

i Polymyxin-based therapy (PBT): Definitive antimicrobial regimens that included polymyxin B or colistin, administered either as monotherapy or in combination with other agents; excluding ceftazidime-avibactam, aztreonam.
ii Non-polymyxin regimens (NPR): The NPR group comprised heterogenous regimens, including β-lactam/β-lactamase inhibitor combinations (with or without aztreonam), carbapenems, tigecycline, aminoglycosides, fosfomycin or combination therapies selected based on in vitro susceptibility, reflecting real-world clinical practice.

When patients received both regimens sequentially, classification was based on the therapy administered for the longer cumulative duration, reflecting the predominant definitive treatment strategy during hospitalization. This approach was selected to capture real-world therapeutic practice, although some degree of exposure misclassification may remain in patients who underwent treatment escalation or de-escalation.

### 2.4 Microbiological procedures

Blood samples were collected under aseptic conditions from peripheral veins or arterial lines and inoculated into BACT/ALERT^®^ culture bottles. Cultures were processed using the BACT/ALERT^®^ VIRTUO^®^ automated microbial detection system. Positive samples were subcultured onto blood and MacConkey agar plates, followed by standard identification through colony morphology, gram staining, and biochemical tests. Final species confirmation was achieved using MALDI-TOF mass spectrometry.

Antibiotic susceptibility testing (AST) was performed using the VITEK 2 Compact system (bioMérieux, Durham, NC, USA) with standard antibiotic panels including amikacin, amoxicillin-clavulanic acid, cefotaxime/ceftriaxone, cefuroxime, ciprofloxacin/ofloxacin, trimethoprim-sulfamethoxazole, gentamicin, aztreonam, cefoperazone-sulbactam, cefpirome/cefepime, imipenem, piperacillin- tazobactam, meropenem, fosfomycin, and ceftazidime-avibactam. For isolates suspected of pan-drug resistance (PDR), defined according to the international expert proposal for standardized definitions of acquired resistance [47], manual synergy testing by disk diffusion was performed to assess potential combination efficacy using established phenotypic synergy-testing approaches [48]. All susceptibility results were interpreted in accordance with the Clinical and Laboratory Standards Institute (CLSI) guidelines [49].

### 2.5 Data collection and follow-up

Patients were followed from the date of first positive CRKP blood culture until hospital discharge or death. The date of initiation of definitive antimicrobial therapy served as the cohort entry point. Clinical and microbiological data were extracted retrospectively from the electronic medical record system. We selected candidate variables based on clinical relevance and prior evidence in sepsis. All the clinical and microbiological data were retrieved from the Medical Records Department of the participating tertiary care hospitals.

Data collected included:

i Demographics and baseline characteristics: age, gender, comorbidities, pre-admission clinical features, vital signs.
ii Severity indices: Acute physiology and chronic health evaluation II (APACHE II), sequential organ failure assessment (SOFA), Charlson comorbidity index (CCI) and Pitts bacteraemia score, calculated at baseline.
iii Inflammatory markers: baseline C-reactive protein (CRP) and procalcitonin levels.
iv Microbiological data: source of infection, prior colonization or infection with CRKP, CRKP isolation at other sites, and presence of co-pathogens.
v Treatment details: type of regimen (PBT or NPR), use of monotherapy or combination therapy, adjunctive antibiotics, total duration of definitive therapy, and time to initiation post-index culture.
vi Clinical outcomes: microbiological clearance, ICU admission, mechanical ventilation, vasopressor requirement, renal replacement therapy and other effectiveness parameters.

### 2.6 Missing data management

Variables with >20% missing values were excluded from analysis. For variables with <20% missing data, median imputation was applied. Implausible or extreme outliers were treated as missing prior to imputation. Details of variables subjected to imputation are provided in S1 Table.

### 2.7 Outcomes measures

The primary effectiveness outcome was all-cause in-hospital mortality. The primary safety outcomes were nephrotoxicity, defined as the development of acute kidney injury or new requirement for dialysis following definitive therapy. Secondary outcomes included clinical cure, microbiological clearance, time to clearance, persistence of CRKP at secondary sites, and the need for ICU care, mechanical ventilation, or vasopressors within three days after initiation of definitive therapy. Resource utilization parameters—such as ICU-free days, ventilator-free days, vasopressor-free days, and antibiotic-free days—were also assessed.

### 2.8 Statistical analysis

All statistical analysis were conducted using Statistical Package for the Social Sciences (SPSS) version 29.0 (IBM Corp. Armonk, NY, USA) [50]. Categorical variables are presented as frequencies and percentages, with group comparisons performed using the chi-square or fisher’s exact test as appropriate. Continuous variables were expressed as mean ± standard deviation (SD) or median (interquartile range, (IQR)), depending on normality determined by the Kolmogorov-Smirnov and Shapiro-Wilk tests. Between group comparisons for non-normally distributed continuous variables were performed using the Mann-Whitney U test. Multicollinearity among independent variables was evaluated using variance inflation factors (VIF) and tolerance (T) values, with VIF > 5 or tolerance< 0.1 indicating collinearity. The primary outcome, i.e., in-hospital mortality, was analyzed using multivariate logistic regression to identify independent predictors. Survival differences between treatment groups were evaluated using Kaplan-Meier survival curves and Cox proportional hazards regression models, with time origin defined as the date of initiation of definitive therapy. Patients discharged alive were censored at the date of discharge or last follow-up. Sensitivity analyses excluding patients who died within 48 hours of initiating therapy were performed to validate robustness of results. To minimize potential immortal time bias related to treatment transitions, time-to-event analyses were anchored to the initiation of definitive therapy and landmark sensitivity analyses excluding early deaths (<48 hours) were performed. Given the observational design and potential for confounding by indication, multivariable modeling was complemented by bootstrap validation and sensitivity analyses; however, propensity score-based methods were not implemented due to sample size limitations and treatment group imbalance.

## 3. Results

### 3.1 Patient cohorts and baseline characteristics

During the study period (January 2019-December 2023), 1,009 patients with *Klebsiella pneumoniae* bacteraemia were identified. Of these, 304 had CRKP bacteraemia. After excluding patients with carbapenem-susceptible *K.pneumoniae* (CSKP) (n = 683), polymicrobial bloodstream infection (n = 13), paediatric or neonatal cases (n = 5), and incomplete records (n = 4), 304 adults with CRKP bacteraemia remained A further 60 patients who died before the initiation of definitive anti-CRKP therapy were excluded, from the comparative outcome analysis, resulting in a final cohort of 244 patients (Fig 1).

These 244 patients were stratified according to definitive antimicrobial regimen into a polymyxin-based therapy (PBT) group (n = 143, 58.6%) and a non-polymyxin regimens (NPR) group (n = 101, 41.4%). Baseline demographic characteristics, including age and sex distribution, as well as most comorbidities, were generally comparable between the two groups (Table 1). The prevalence of chronic liver disease was comparable between the two groups (10.5% vs. 9.9%; p = 0.001), whereas chronic lung disease was more common in the NPR group (5% vs. 3.5%; p = 0.025). Patients receiving PBT had higher baseline disease severity, reflected by significantly higher CCI (4 [0–8] vs. 3 [0–10]; p = 0.025), SOFA score (4 [0–12] vs. 3 [0–12]; p < 0.001), and a significantly higher distribution of Pittsburgh Bacteraemia Score (0 [0–8] vs. 0 [0–7]; p = 0.001). Baseline inflammatory markers were broadly similar, although median procalcitonin levels (6 [0.45–574.20] vs. 6 [0.5–260.10]; p = 0.015) were higher in the PBT group. AST resistance patterns were comparable between both the groups. Patient receiving polymyxin-based therapy had higher baseline disease severity, suggesting preferential use of polymyxins in more critically ill patients, consistent with real-world prescribing patterns. Full baseline characteristics are summarised in Table 1.

**Table 1:** Comparison of baseline characteristics of patients among both the treatment groups.

*Variables*	*Non-polymyxin regimens(n = 101) [n(%)]*	*Polymyxin based therapy (n = 143) [n(%)]*	*p-value*
Age > 65 years	25 (24.8)	43 (30.1)	0.065*
Gender (Male)	75 (74.3)	101 (70.6)	0.756*
Type II Diabetes Mellitus	51 (50.5)	73 (51)	0.841*
Hypertension	47 (46.5)	71 (49.7)	0.375*
Coronary artery disease	21 (20.8)	26 (18.2)	0.115*
Chronic liver disease	10 (9.9)	15 (10.5)	0.001*
Chronic kidney disease	13 (12.9)	24 (16.8)	0.324*
Chronic lung disease	5 (5)	5 (3.5)	0.025*
Carcinoma	25 (24.8)	34 (23.8)	0.745*
Baseline creatinine [median]	1.18 (0.33-8.84)	1.36 (0.41-15.20)	0.12*
APACHE II [median]	12 (0-35)	13 (3-44)	0.345**
CCI [median]	3 (0-10)	4 (0-8)	0.025**
SOFA [median]	3 (0-12)	4 (0-12)	<0.001**
Pitts bacteremia [median]	0 (0-7)	0 (0-8)	0.001**
Baseline CRP [median]	114 (2.32-350)	114 (1.33-376.87)	0.251**
Baseline Procalcitonin [median]	6 (0.5-260.10)	6 (0.45-574.20)	0.015**
AST resistance patterns			
Amikacin	63 (62.3)	93 (65)	
Amoxicillin-clavulanic acid	99 (98.01)	134 (93.7)	
Ampicillin/amoxicillin	100 (99)	135 (94.4)	
Cefotaxime/ceftriaxone	101 (100)	136 (95.1)	
Cefuroxime	101 (100)	135 (94.4)	
Ciprofloxacin/ofloxacin	100 (99)	141 (98.6)	
TMP-SMX	82 (81.1)	121 (84.6)	
Gentamicin	83 (82.1)	118 (82.5)	
Cefoperazone-sulbactam	99 (98)	141 (98.6)	
Cefpirome/cefepime	99 (98.01)	142 (99.3)	
Piperacillin/Tazobactam	101 (100)	142 (99.3)	

**Mann-Whitney U test, *Chi-square test, APACHE II- acute physiology and chronic health evaluation, SOFA- sequential organ failure assessment, CCI- Charlson’s comorbidity index, AST- antibiotic susceptibility testing.

### 3.2 Comparative clinical characteristics and treatment outcomes

Clinical characteristics and outcomes by treatment group are presented in Table 2. Overall, patients treated with PBT experienced substantially worse clinical outcomes than those receiving NPR. With respect to effectiveness, the clinical cure rates were markedly lower in the PBT group compared with the NPR group (15.4% vs. 58.4%; p = 0.02) and in-hospital mortality was significantly higher among PBT recipients (68.5% vs. 38.6%; p < 0.001). The non-polymyxin regimen group included a diverse range of susceptibility-guided therapies, with only a subset of patients receiving ceftazidime-avibactam-based combinations,

**Table 2:** Clinical characteristics and outcomes of patients among both the treatment groups.

*Variables*	*Non-polymyxin regimens (n = 101)*	*Polymyxin based therapy (n = 143)*	*p-value*
Acute kidney injury after definitive therapy	29 (28.7)	80 (55.9)	0.008*
Definitive monotherapy	42 (40.6)	35 (25.1)	0.095*
Two drug combination therapy	51 (50.5)	80 (55.9)	0.576*
More than two drug combination therapy	8 (6.9)	28 (20.3)	0.399*
Adjunctive antibiotics other than aztreonam			0.490*
Monotherapy	28 (27.7)	79 (55.2)	
Dual therapy	9 (8.9)	20 (14)	
Duration of definitive therapy [median]	6 (2-19)	6 (3-16)	0.021**
Antibiotic free days	23 (22.8)	19 (13.3)	0.004*
Requirement of dialysis after definitive therapy	60 (59.4)	112 (78.3)	<0.001*
Clinical cure	59 (58.4)	22 (15.4)	0.02*
In hospital mortality	39 (38.6)	98 (68.5)	<0.001*
Microbiological clearance	46 (45.5)	54 (37.8)	0.141*
Time to microbiological clearance [median]	0 (0-22)	0 (0-32)	0.670**
Requirement of mechanical ventilation ≥3 days after definitive therapy	46 (45.5)	96 (67.1)	<0.001*
Requirement of vasopressors ≥3 days after definitive therapy	30 (29.7)	76 (53.1)	0.01*
Requirement of ICU stay after starting definitive therapy	70 (69.3)	111 (77.6)	0.003*
Infection site of CRKP apart from blood	51 (50.5)	71 (49.7)	0.004*
Pathogenic bacteria other than CRKP in blood	21 (20.8)	34 (23.8)	0.034*
Duration of hospitalization [median]	16 (4-50)	19 (4-119)	0.008**
Vasopressor free days [median]	15 (0-50)	16 (0-118)	0.17**
Ventilator free days [median]	10 (0-50)	12 (0-98)	0.059**
ICU free days [median]	7 (0-50)	5 (0-41)	0.319**
Time to antibiotic initiation from index culture [median]	2 (0-4)	2 (0-4)	0.36**

**Mann-Whitney U test, *Chi-square test.

Renal outcomes were also less favourable among patients receiving PBT, although kidney injury in critically ill patients is likely multifactorial. Acute kidney injury (AKI) following definitive therapy occurred more frequently in the PBT group than in the NPR group (55.9% vs. 28.7%; p = 0.008), and the requirement for dialysis after definitive therapy was significantly higher with PBT (78.3% vs. 59.4%; p < 0.001). Although the median duration of definitive therapy was similar (6 [3–16] days vs. 6 [2–19] days; p = 0.021), patients in the PBT group had fewer antibiotic-free days than those in the NPR group (13.3% vs. 22.8%; p = 0.004). The total duration of hospitalization was also longer in the PBT group (19 [4–119] vs 16 [4–50] days; p = 0.008). Need for organ support and intensive care after initiation of definitive therapy was consistently higher in the PBT cohort. Compared with NPR, PBT was associated with more frequent mechanical ventilation for ≥3 days (67.1% vs. 45.5%; p < 0.001), vasopressor requirement for ≥3 days (53.1% vs. 29.7%; p = 0.01), and requirement of ICU admission after starting definitive therapy (77.6% vs. 69.3%; p = 0.003).

Microbiological outcomes were broadly similar between the two groups. Rates of microbiological clearance did not differ significantly (45.5% with NPR vs. 37.8% with PBT; p = 0.141), and median time to clearance was comparable (p = 0.670). The presence of pathogenic bacteria other than CRKP in blood cultures (23.8% vs. 20.8%; p = 0.034) and CRKP at non-bloodstream sites (49.7% vs. 50.5%; p = 0.004) was more frequently observed among patients in the PBT group. The total duration of hospitalization was longer in the PBT group (19 [4–119] days vs. 16 [4–50] days; p = 0.008). The baseline clinical characteristics and treatment outcomes of the NPR and PBT groups are detailed in Table 2. A detailed summary of the most frequently used definitive antimicrobial regimens in both treatment groups is provided in S2 Table.

### 3.3 Risk factor analysis for in-hospital mortality

Factors associated with in-hospital mortality among patients with CRKP bacteraemia are shown in Table 3. On univariate cox regression, several variables were significantly associated with increased risk of death, including chronic liver disease, AKI after initiation of therapy, higher SOFA score, treatment with a polymyxin-based definitive regimen, requirement of dialysis, prolonged mechanical ventilation (≥3 days), vasopressor use for ≥3 days, ICU stay after definitive therapy, and the presence of additional pathogenic bacteria in blood. In contrast, a greater number of antibiotic-free days and primary CRKP BSI (vs. secondary bacteraemia) were associated with a lower risk of in-hospital mortality.

**Table 3:** Univariate and multivariate Cox regression analysis to predict factors associated with in-hospital mortality in CR-KP patients.

*Variables*	*Univariate analysis*		*Multivariate analysis*	
	*P value*	*HR (95% CI)*	*P value*	*aHR (95% CI)*
Age	0.55	0.895 (0.618-1.295)		
Male gender	0.889	0.974 (0.669-1.418)		
Type II Diabetes mellitus	0.983	0.996 (0.713-1.393)		
Hypertension	0.727	0.942 (0.673-1.318)		
Chronic liver disease	0.011	1.853 (1.151-2.982)	0.02	2.31 (1.884-3.642)
Chronic kidney disease	0.476	1.174 (0.755-1.826)		
Acute kidney injury after initiation of therapy	<0.001	1.814 (1.291-2.547)		
APACHE II	0.352	1.01 (0.989-1.032)		
CCI	0.109	1.057 (0.988-1.032)		
SOFA	0.024	1.075 (1.01-1.145)	0.027	1.514 (1.285-1.928)
Pitts bacteremia	0.213	1.056 (0.969-1.151)		
Treatment with polymyxins as definitive therapy	0.02	1.78 (1.42-2.85)	0.015	1.595 (1.392-1.903)
Antibiotic free days	<0.001	0.165 (0.073-0.374)		
Requirement of dialysis after definitive therapy	0.01	1.559 (1.112-2.184)	0.048	3.11 (1.008-4.59)
Primary BSI CR-KP	0.018	0.613 (0.408-0.92)		
Requirement of mechanical ventilation ≥3days after definitive therapy	<0.001	3.478 (2.281-5.305)		
Requirement of vasopressors ≥3days after definitive therapy	<0.001	2.984 (2.097-4.246)		
Requirement of ICU stay after starting definitive therapy	<0.001	3.278 (1.885-5.701)	0.02	1.474 (1.252-1.891)
Pathogenic bacteria other than CRKP in blood	0.022	1.53 (1.063-2.203)		

**Fig 2:**
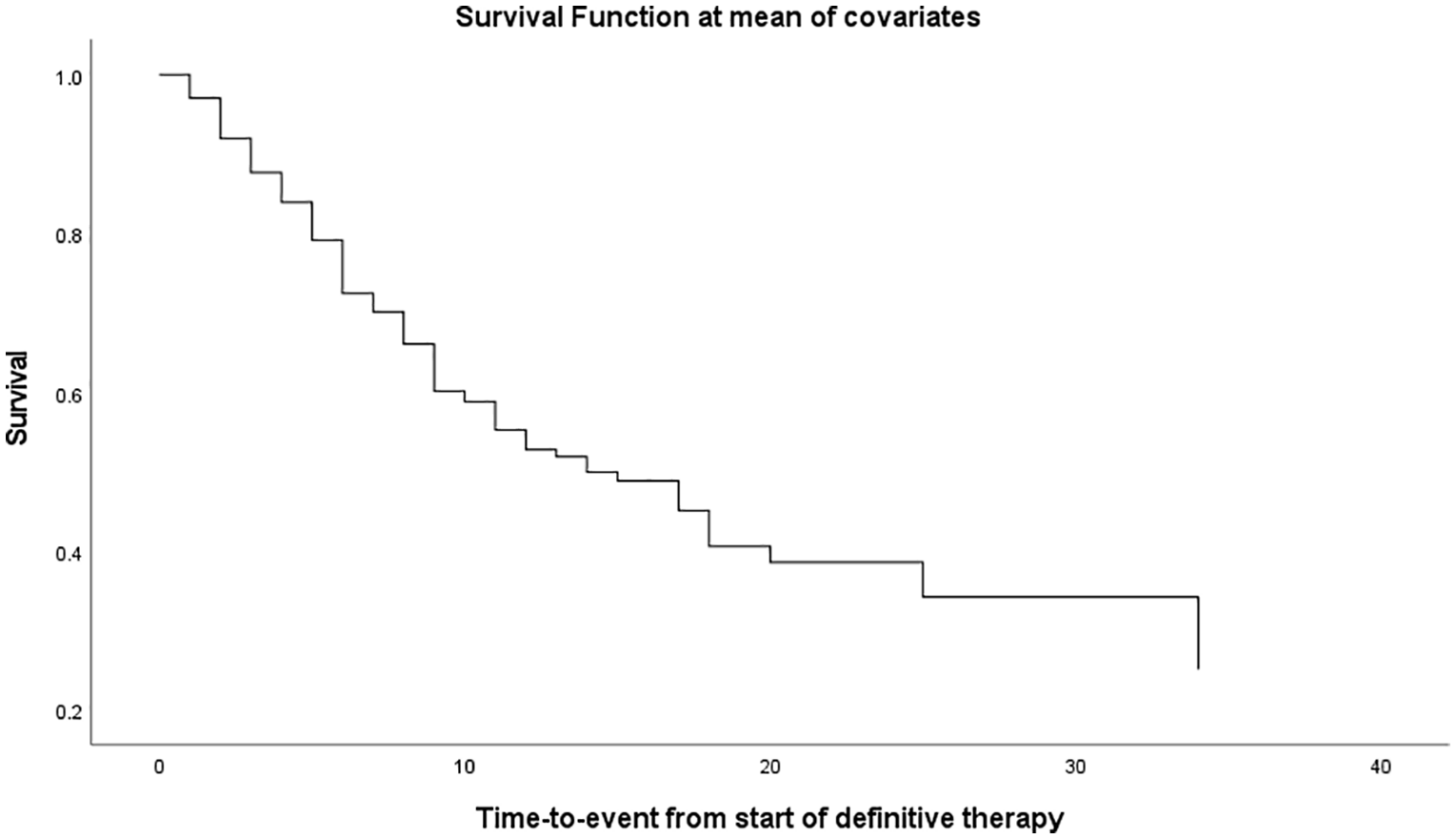
Adjusted survival function derived from the multivariable Cox proportional hazards model among patients with CR-KP infection.

In the multivariable Cox proportional hazard model, five variables remained independently associated with in-hospital mortality: treatment with polymyxins as definitive therapy (aHR: 1.595; 95% CI: 1.392–1.903; p = 0.015), higher SOFA score (aHR: 1.514; 95% CI: 1.285–1.928; p = 0.027), presence of chronic liver disease (aHR: 2.31; 95% CI: 1.884–3.642; p = 0.02), requirement of dialysis following definitive therapy (aHR: 3.11; 95% CI: 1.008–4.59; p = 0.048), and ICU stay after initiation of definitive therapy (aHR: 1.474; 95% CI: 1.252–1.891; p = 0.02). Adjusted hazard ratios (aHR) and 95% CI for these predictors are detailed in Table 3. The adjusted survival function derived from the multivariable Cox model (Fig 2), depicts the predicted survival probability for a patient with average covariate values. The curve shows a steep decline in survival during the first 10 days following initiation of definitive therapy, followed by a more gradual plateau, thereafter, reflecting substantial early mortality and persistent residual risk among survivors. The model-estimated 30-day survival probability for an average-risk patient was approximately 35–40%.

To assess model stability, bootstrap validation (979 samples) was performed (Table 4). The bootstrap analysis confirmed the robustness of the main predictors, with treatment with polymyxin-based therapy (p = 0.018), higher SOFA score (p = 0.008), chronic liver disease (p = 0.039), dialysis requirement (p = 0.012), and ICU stay (p = 0.02) after therapy initiation consistently associated with mortality. To explore the influence of early deaths and potential immortal time bias, a landmark sensitivity analysis excluding patients who died within 48 hours of starting definitive therapy (n = 210) was conducted (Table 5). In this restricted cohort, treatment with polymyxins, higher SOFA, dialysis requirement, and prolonged vasopressor use remained independently associated with in-hospital mortality, while the effect of chronic liver disease appeared to be more prominent in the early mortality phase.

**Table 4:** Bootstrap multivariable regression analysis after multivariate analysis to predict factors associated with in-hospital mortality among CR-KP patients (n = 979 samples).

*Variables*	*Bias*	*P value*	*BCa 95% CI*
Chronic liver disease	−0.023	0.039	−0.775-0.272
SOFA	0.07	0.008	0.063-0.085
Treatment with polymyxins as definitive therapy	−0.519	0.018	−0.971-0.103
Requirement of dialysis after definitive therapy	1.135	0.012	1.043-2.273
Requirement of ICU stay after starting definitive therapy	−0.747	0.02	−1.403-0.132

**Table 5:** Sensitivity analysis for factors associated with in-hospital mortality excluding patients who died within 48hrs after initiating definitive therapy (landmark analysis) [n = 210].

*Variables*	*P value*	*HR (95% CI)*	*P value*	*aHR (95% CI)*
Chronic liver disease	0.021	1.229 (1.135-1.361)	0.032	1.36 (1.102-2.547)
CCI	0.035	1.175 (1.002-1.293)		
SOFA	0.005	1.351 (1.158-2.642)	0.041	1.874 (1.475-2.189)
Pitts bacteremia	0.013	1.42 (1.154-1.85)		
Treatment with polymyxins as definitive therapy	0.028	2.12 (1.64-2.78)	0.003	2.13 (1.561-2.891)
Requirement of dialysis after definitive therapy	0.047	2.75 (1.58-4.75)	0.014	1.571 (1.28-2.132)
Requirement of mechanical ventilation ≥3days after definitive therapy	0.006	1.542 (1.032-2.015)		
Requirement of vasopressors ≥3days after definitive therapy	0.038	1.871 (1.560-2.355)	0.032	1.89 (1.127-2.974)
Requirement of ICU stay after starting definitive therapy	0.023	1.472 (1.252-1.891)		

### 3.4 Survival and microbiological clearance

**Fig 3:**
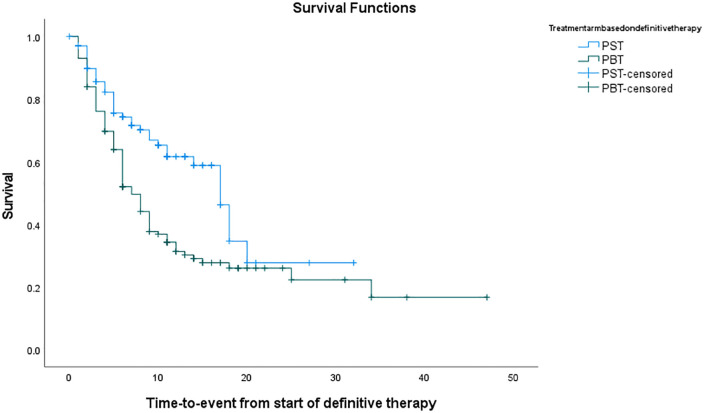
Kaplan-Meier survival curves comparing in-hospital mortality among patients with CRKP infection treated with polymyxin based versus non-polymyxin regimens [log rank test = 0.001].

Kaplan-Meier survival curves comparing in-hospital mortality between PBT and NPR are depicted in Fig 3. A clear separation between the curves was observed, with a higher cumulative probability of survival in the NPR group throughout the follow-up period. The log-rank (Mantel–Cox) test demonstrated a statistically significant difference in survival distributions between PBT and NPR (χ² = 11.161, p < 0.001), which was corroborated by the Breslow (χ² = 11.531, p < 0.001) and Tarone-Ware tests (χ² = 12.430, p < 0.001). In summary, Kaplan-Meier survival curves revealed superior survival among NPR recipients (log-rank p < 0.001).

**Fig 4:**
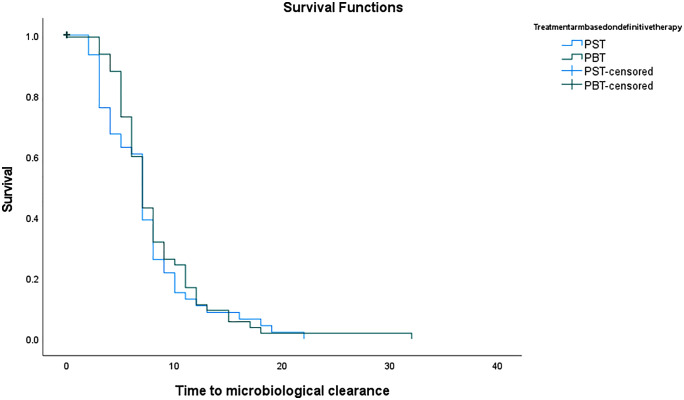
Kaplan-Meier curves comparing time to microbiological clearance among patients with CRKP infection treated with polymyxin-based and non-polymyxin regimens [log rank (Mantel-Cox) =0.57].

Time to microbiological clearance, assessed by Kaplan-Meier analysis, is presented in Fig 4. The curves for PBT and NPR were largely overlapping, and no significant difference in time to clearance was observed (log-rank χ² = 0.322, p = 0.570), consistent with alternative test statistics, including the Breslow (Generalized Wilcoxon) test (χ² = 0.554, p = 0.457) and the Tarone–Ware test (χ² = 0.534, p = 0.465).

**Fig 5:**
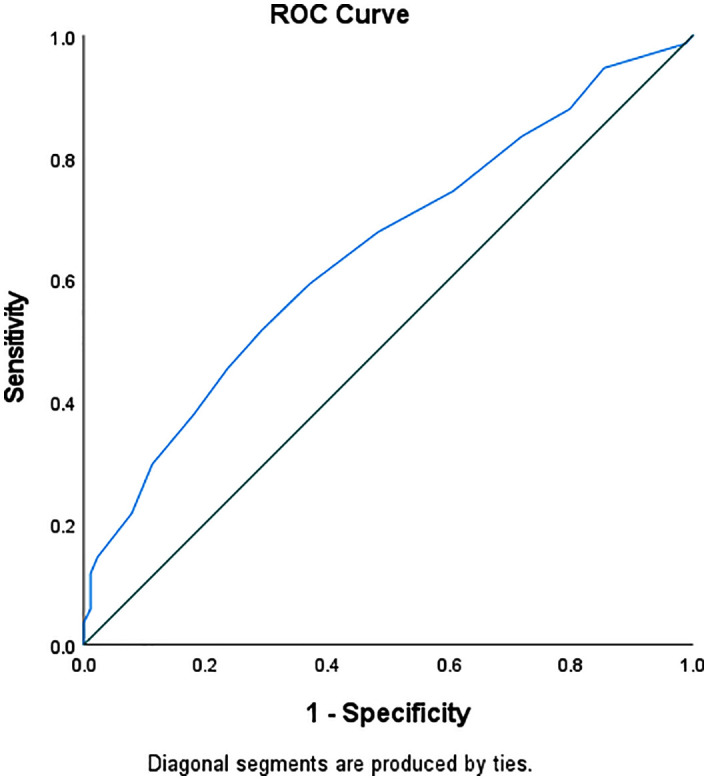
ROC curve of SOFA score to predict in-hospital mortality in CRKP patients.

Receiver operating characteristic (ROC) curve analysis was conducted to assess the discriminative ability of SOFA score in predicting in-hospital mortality. The analysis showed an AUC of 0.65, P = 0.00, 95% CI (0.62–0.75) for SOFA score with 67.5% sensitivity and 62% specificity. Notably, the cut-off score of SOFA was noted down at 6 (Fig 5).

## 4. Discussion

CRKP bacteraemia remains a major clinical and public-health concern due to limited therapeutic options and high mortality [2,19,51]. In this multicentre, five-year retrospective cohort study, we compared polymyxin-based therapy with non-polymyxin regimens for CRKP bacteraemia. Three principal findings emerged:

i PBT was associated with significantly poor clinical outcomes, including low clinical cure and high in-hospital mortality.
ii PBT resulted in substantially greater nephrotoxicity and need for organ support despite similar microbiological clearance between groups.
iii Treatment with polymyxins, higher SOFA score, chronic liver disease, dialysis requirement, and ICU stay after therapy initiation independently predicted mortality.

These results suggest that non-polymyxin regimens may be associated with more favourable clinical outcomes and safety profiles compared with polymyxin-based therapy whenever feasible. The NPR cohort reflected the real-world evolution of CRKP management between 2019 and 2023, spanning the introduction of ceftazidime-avibactam plus aztreonam (CAZ-AVI + AZM) into clinical practice. Only one-quarter of NPR recipients received CAZ-AVI + AZM, while the remainder were treated with other active regimens, including high-dose carbapenems, tigecycline, aminoglycosides, fosfomycin or other β-lactams according to susceptibility results. The NPR group encompassed a heterogenous, range of antimicrobial strategies, reflecting real-world practice in low- and middle-income settings, where treatment is individualized based on susceptibility patterns and drug availability. Therefore, the observed association between NPR and improved outcomes should be interpreted cautiously and should not be assumed to reflect a uniform benefit across all NPR therapeutic combinations. Further prospective studies are required to evaluate the comparative effectiveness of individual regimen strategies.

Our findings align with growing international evidence demonstrating superior outcomes with CAZ-AVI-based therapy with polymyxin-containing regimens for CRKP bloodstream infections. Several meta-analyses and multicentre studies have shown higher clinical success and lower 28–30-day mortality with CAZ-AVI, including reductions of 15–20% points in mortality compared with polymyxins [19,52,53]. Indian cohorts similarly report significantly lower odds of death and nephrotoxicity with CAZ-AVI (often combined with aztreonam) relative to polymyxin B or colistin [23,42]. The present study extends these observations by demonstrating that, even when CAZ-AVI use is limited, a non-polymyxin regimen approach, built on susceptibility-guided combinations of available agents, confers clear survival and safety benefits in a high-burden, low-resource setting.

The superior outcomes observed with NPR likely reflect the use of agents possessing more predictable PK/PD characteristics and a broader therapeutic window. Optimized dosing of carbapenems, fosfomycin and aminoglycoside use in combination may all enhance bacterial killing while reducing host toxicity [14,19,27,36]**.** In contrasts, polymyxins exhibit variable plasma exposure, poor penetration at infection sites, and a narrow margin between efficacy and toxicity, leading to frequent treatment-related complications. This therapeutic disadvantage is magnified in critically ill patients, where achieving target concentrations without inducing nephrotoxicity is particularly challenging [23].

Higher rates of AKI were observed among patients receiving PBT; however, renal dysfunction in critically ill patients with CRKP bacteraemia is multifactorial and may be influenced by illness severity, hemodynamic instability, vasopressor exposure, ICU-related factors, and concomitant nephrotoxic therapies. More than half of patients receiving PBT developed AKI, and dialysis was required in nearly four-fifths (55.9% vs 28.7%) of patients—rates comparable to or exceeding those reported in other polymyxin cohorts. By contrast, nephrotoxicity rates of 10–30% have been reported with CAZ-AVI-based regimens [53–56]. Although these findings are consistent with the recognized nephrotoxic profile of polymyxins, the retrospective design precludes definitive attribution of causality. [57]. AKI may contribute to prolonged hospitalization, treatment interruption, dosing challenges, and multi-organ dysfunction, potentially explaining the fewer antibiotic-free days and longer hospital stay observed among PBT recipients [58].

Interestingly, microbiological clearance rates and time of clearance were similar between groups. Previous studies have reported higher eradication with CAZ-AVI [19,44], but our data suggest that mortality differences are not primarily due to failure of bacterial clearance. Instead, PBT appears to impose a greater “pathophysiological cost” of therapy. PBT patients experienced higher AKI rates, more frequent mechanical ventilation and vasopressor requirements, and greater prevalence of disseminated CRKP infection or co-pathogen bacteraemia, all indicative of clinical instability. Thus, even when microbiological success is achieved, polymyxin toxicity and its systemic consequences likely undermine survival. NPR, conversely, enabled comparable bacterial control with less collateral organ damage, translating into improved outcomes. However, interpretation of ceftazidime-avibactam-based outcomes should be cautious in the absence of molecular confirmation of underlying carbapenemase mechanisms.

Multivariable analysis identified five independent predictors of in-hospital mortality. Treatment with polymyxin-based therapy remained independently associated with mortality even after adjustment for illness severity [33]. However, patients receiving polymyxins exhibited greater baseline illness severity, as reflected by higher SOFA, CCI, and Pitts bacteraemia scores, and residual confounding related to disease severity may have partially contributed to the observed mortality differences. Higher SOFA scores predicted mortality as a marker of cumulative organ dysfunction, consistent with established sepsis prognostic models [24]. Chronic liver disease conferred additional risk, reflecting impaired host defence and metabolic reserve [59]. Dialysis requirement and ICU stay after therapy initiation reflected clinical deterioration and escalating organ support, both previously linked to adverse outcomes in CRKP bacteraemia [60].

Internal bootstrap validation confirmed the robustness of these associations, and landmark sensitivity analysis excluding early deaths demonstrated that these variables continued to predict later mortality. collectively, these analyses strengthen causal inference that both baseline severity and treatment choice influence survival trajectories in CRKP bacteraemia. The model-derived survival function further illustrates the dynamic nature of risk. The steep decline in survival within the first 10 days after definitive therapy underscores the lethality of CRKP bacteraemia and the urgency of timely, optimised antimicrobial intervention. Even beyond this acute phase, survival probabilities remained low—approximately 35–40% at 30 days for an average-risk patient—highlighting the sustained mortality burden associated with these infections despite modern therapies. Accordingly, treatment-related associations should be interpreted cautiously within the context of dynamic real-world antimicrobial prescribing practices.

### 4.1 Strengths and clinical implications

This study offers several notable strengths. It represents one of the largest multicentre evaluations of CRKP bacteraemia, encompassing a five-year period during which treatment paradigms rapidly evolved. This temporal breadth captures the transition from polymyxin-based regimens to non-polymyxin combinations, providing a comprehensive view of real-world practice. In addition, the analysis incorporated multiple complementary methods to ensure robustness and minimize bias.

Clinically, these findings provide supportive observational evidence favouring polymyxin-sparing approaches for CRKP bacteraemia where appropriate alternatives are available. The consistent signal of excess mortality and nephrotoxicity with PBT supports re-evaluation of polymyxin’s role as definitive therapy. Non-polymyxin regimens—particularly CAZ-AVI ± AZM in regions with Metallo-β-lactamase co-production—may represent promising therapeutic alternatives, although prospective comparative studies remain necessary. In settings where newer β-lactam/β-lactamase inhibitors remain inaccessible; clinicians should maximise available non-polymyxin agents through tailored combination strategies guided by susceptibility testing. Additionally, identification of high-risk clinical profiles can facilitate timely escalation of care and targeted stewardship interventions. These findings should therefore be interpreted within the context of potential residual confounding, although the consistency across multiple analytical approaches supports the robustness of the observed associations. These insights are directly relevant for antimicrobial stewardship programmes in low- and middle-income countries, where polymyxins are still widely used due to cost and availability.

### 4.2 Limitations and future direction

This study has several limitations. First, its retrospective design introduces the potential for confounding by indication, as patients with greater baseline illness severity were more likely to receive polymyxin-based therapy. Although we adjusted for key clinical confounders using multivariable cox regression and performed bootstrap validation and landmark sensitivity analyses, residual confounding cannot be excluded. Therefore, the observed association between polymyxin-based therapy and increased mortality should be interpreted as associative rather than casual. While propensity score-based methods such as matching or inverse probability weighting may further reduce bias, their application was limited by sample size constraints and treatment imbalance, which could have reduced statistical power and generalizability.

Second, the higher incidence of AKI observed in the polymyxin group should be interpreted cautiously, as AKI in critically ill patients is multifactorial and may reflect the combined effects of sepsis severity, hemodynamic instability vasopressor exposure, ICU-related factors and concomitant nephrotoxic medications. Consequently, a direct causal relationship between polymyxin exposure and AKI cannot be established.

Third, molecular characterization of carbapenem resistance mechanisms (e.g. *bla*NDM, *bla*OXA-48, *bla*KPC*) was not routinely available across participating centres, precluding genotypic-specific analyses. Given that the efficacy of newer agents such as ceftazidime-avibactam is influenced by the underlying carbapenemase mechanism, the absence of molecular data limits interpretation of regimen-specific effectiveness. Treatment decisions in this study were therefore based primarily on phenotypic susceptibility profiles and institutional stewardship practices.

Fourth, the non-polymyxin regimen group comprised heterogenous antimicrobial strategies, and subgroup analyses were not feasible because of limited sample size and unequal distribution of specific regimens. Consequently, regimen-specific effects could not be reliably evaluated. Additional limitations include the use of median imputation for variables with limited missingness, which may have introduced minor bias, and potential exposure misclassification among patients receiving sequential polymyxin and non-polymyxin therapies. Although landmark sensitivity analyses were performed to mitigate immortal time bias, a formal time-dependent exposure analysis was not feasible because of heterogenous switching patterns and sample size limitations.

Future research should prioritise perspective, ideally randomised, comparisons of specific non-polymyxin regimens—particularly CAZ-AVI ± AZM—against polymyxin-containing therapy for CRKP bacteraemia. Integration of rapid molecular diagnostics with clinical outcome assessment would enable genotypic-guided therapeutic evaluation and improve understanding of regimen-specific effectiveness. Larger cohorts should also incorporate severity-stratified or propensity-based analyses to better delineate treatment effects across different levels baseline clinical severity. Furthermore, real-world pharmacovigilance and PK/PD studies are needed to better characterize toxicity profiles and optimize dosing of both established and emerging therapies in critically ill populations. Stewardship-focused strategies will remain essential to ensure sustainable use of newer antimicrobial agents, particularly in LMIC settings where inappropriate use may accelerate resistance development.

## 5. Conclusion

In conclusion, this multicentric retrospective cohort study demonstrated that non-polymyxin regimens were associated with higher clinical cure rates and lower risks of acute kidney injury and in-hospital mortality compared with polymyxin-based regimens in patients with carbapenem-resistant *K. pneumonia*e bacteraemia. Given the observational nature of the study and potential residual confounding, these findings should be interpreted cautiously and viewed as hypothesis-generating rather than causal. Nevertheless, the results contribute important real-world evidence supporting further evaluation of polymyxin-sparing therapeutic strategies for CRKP bacteraemia particularly in critically ill populations.

## Supporting information

S1 Table Missing number for included variables in the dataset. (DOCX)

S2 Table Most frequently administered definitive antimicrobial regimens among patients receiving polymyxin-based therapy and non-polymyxin regimens. (DOCX)

## Abbreviations

MDR-GNB: Multidrug-resistant Gram-negative bacteria
WHO: World Health Organization
CRE: Carbapenem-resistant *Enterobacterales*
CPE: Carbapenemase-producing *Enterobacterales*
CRKP: Carbapenem-resistant *Klebsiella pneumoniae*
ICMR: Indian Council of Medical Research
UTIs: Urinary tract infections
IDSA: The Infectious Diseases Society of America
CAZ-AVI+AZM: Ceftazidime-avibactam plus aztreonam
MBL: Metallo-β-lactamase
PBT: Polymyxin-based therapy
NPR: Non-polymyxin regimens
APACHE II: Acute physiology and chronic health evaluation II
SOFA: Sequential organ failure assessment
CCI: Charlson comorbidity index
CRP: C-reactive protein
CSKP: Carbapenem sensitive *Klebsiella pneumoniae*
AKI: Acute kidney injury
ICU: Intensive-care unit
CLSI: Clinical and Laboratory Standards Institute
ROC: Receiver operating characteristic
aHR: Adjusted Hazard Ratio
AST: Antibiotic susceptibility test
