# Supplementary material for: Comparative clinical outcomes of polymyxin-based versus non-polymyxin regimens as definitive therapy in Carbapenem-resistant Klebsiella pneumoniae bacteraemia
Source: PLoS One. 2026 Jul 15;21(7):e0353799. doi: 10.1371/journal.pone.0353799 (PMC13372112; doi:10.1371/journal.pone.0353799)
Supplement: S1 Table — (DOCX) [file pone.0353799.s001.docx]

***Supplementary table SI: Missing number for included variables in the dataset***

| ***Variables*** | ***Missing, n (%), (n=244)*** |
| --- | --- |
| T. bilirubin | 12 (4.9%) |
| Sr. Creatinine | 8 (3.2%) |
| C-Reactive Protein | 7 (2.8%) |
| Procalcitonin | 15 (6.14%) |
| WBC | 9 (3.68%) |
