# Supplementary material for: Comparative clinical outcomes of polymyxin-based versus non-polymyxin regimens as definitive therapy in Carbapenem-resistant Klebsiella pneumoniae bacteraemia
Source: PLoS One. 2026 Jul 15;21(7):e0353799. doi: 10.1371/journal.pone.0353799 (PMC13372112; doi:10.1371/journal.pone.0353799)
Supplement: S2 Table — (DOCX) [file pone.0353799.s002.docx]

***Supplementary Table SII: Most frequently administered definitive antimicrobial regimens among patients receiving polymyxin-based therapy and non-polymyxin regimens***

| ***Antibiotics*** | ***Non-polymyxin regimens(n=101) [n(%)]*** | ***Polymyxin based therapy (n=143) [n(%)]*** |
| --- | --- | --- |
| Polymyxin B + Amikacin | 0 | 2 |
| Colistin + Tigecycline | 0 | 7 |
| Imipenem + Tigecycline | 2 | 0 |
| Meropenem +Tigecycline | 6 | 0 |
| Meropenem + Teicoplanin | 3 | 0 |
| Polymyxin B + Tigecycline | 0 | 32 |
| Polymyxin B + Meropenem | 0 | 3 |
| Polymyxin B + Teicoplanin | 0 | 2 |
| Colistin + Meropenem | 0 | 2 |
| Cefoperazone + Sulbactam | 3 | 0 |
| Fosfomycin + Tigecycline | 2 | 0 |
| Colistin + Meropenem | 0 | 2 |
| Polymyxin B + Fosfomycin | 0 | 3 |
| Ceftazidime-avibactam + aztreonam | 21 | 0 |
